# Editorial: Prevention, diagnosis and treatment of rare disorders

**DOI:** 10.3389/fphar.2022.1026064

**Published:** 2022-09-28

**Authors:** Timothy M. Cox, Anna Tylki-Szymańska, Ségolène Aymé, Marc Dooms

**Affiliations:** ^1^ University of Cambridge, Cambridge, United Kingdom; ^2^ Children’s Memorial Health Institute (IPCZD), Warsaw, Poland; ^3^ Institut National de la Santé et de la Recherche Médicale (INSERM), Paris, France; ^4^ University Hospitals Leuven, Leuven, Belgium

**Keywords:** rare diseases, orphan drugs, global, venture capitalism, prevention, diagnosis, treatment, equitable access

We gratefully celebrate the quality and far-reaching geographical representation of the articles in this volume, “Prevention, Diagnosis and Treatment of Rare Disorders”, commissioned for *Frontiers in Pharmacology*. At the time of writing, 19 articles have attracted nearly 60,000 reads. The portfolio reveals an impressive commitment by more than 70 authors with diverse professional backgrounds, excluding the many dedicated reviewers who, beyond matters of validity, have together ensured freshness and originality. This is no easy matter: after centuries in obscurity, exponential investment in the field of rare diseases has attracted intense interest as well as scrutiny from many corners (Tsigkos et al., 2021). Here we briefly review the status of this now vast field and its central mission. Innumerable people are afflicted by countless rare diseases without access to expertise or effective treatments and so the humanitarian mandate remains clear. However, immense opportunities, still exist to build on the achievements of the last four decades. One key to the future will surely be imaginative exploration and diversification of funding models realistically to advance – and equitably share – the benefits that accompany the healing concept in rare diseases.

## Retrospect

To maintain the focus on those who suffer from rare diseases, it is necessary to distinguish the enabling aspects of the legislation that has incentivized drug development, from the real world of practice. The birth of the commercial edifice is well known. Its conception, about 40 years ago, was the work of the National Organization of Rare Disorders - a loose 1970s coalition of supporters, advocates and families of patients with rare diseases in the US. The demand was for new legislation to support development of drugs for treating rare diseases–“orphan” drugs for orphan diseases – an apt, if emotional term, now largely replaced by “rare”. The Orphan Drug Act, 97/414 (ODA), was introduced by the United States Congress in 1983. Singapore followed the initiative in 1991; Japan in 1993 and Australia, in 1998. In 2000 the European Commission introduced Regulation EC 141/2000 (European Medicines Agency, 2000) for its now 28 or so constituent countries.

We now see that the ODA was a gigantic and influential capitalistic experiment! Imperfect it may be, but the concept is applied publicly and liberally in the United States, Western Europe, Australasia and parts of South America, Scandinavia and elsewhere. The intention was to bring effective treatments to patients with diseases so rare that without radical measures, there would be little or no commercial justification for the costs. Human intentions, are often expressed as hopes–perhaps, psychologically speaking, to invest them with good fortune. Such has been the pharmaceutical success of this initiative, that the marketing exclusivity, tax-breaks and other support (including support “in kind”) obviated the need for luck. The incentives proved to be real: spectacular commercial realities (profit) and translational research discoveries emerging from development of drugs and devices for rare diseases have exceeded expectations (Aartsma-Rus et al.). The market for the treatment of rare diseases was more than $144 billion in 2019 and annual growth exceeds 10%.

The iniquitous scourge of patients with rare diseases who, for one reason or another, were denied access to critical therapies should now be a matter for the past. That is, in the relatively rich developed Western-style political economies. In countries where Orphan Drug legislation is in place, the access problem based on unmet needs and drug availability, is coming to an end. Nature has indeed been “generous in her senseless experiments on mankind” (Koestler, 1941). The material and financial resources accompanying the vast present-day pantheon of Biotech, armed by the 1983 US Orphan Drug Legislation, followed by numerous international imitations - and realised by judicious investment in translational science - has radically changed perceptions and hopes for patients, companies and physicians. Elsewhere, the inequalities of access persist, indeed widen; and whole nations representing billions of people are affected. It is difficult not to feel hypocritical shame that the extremity of needs that may never be met in one region are already met in another.

It is not all gloom: the pharmaceutical revolution has brought much needed general benefits and supportive recognition for patients in this field. One of the first was Orphanet (Orphanet, 2022): established in France by the National Institute for Health and Medical Research (INSERM) in 1997 and supported by grants awarded by the European Commission, this was incorporated as a European endeavour 3 years later. The aim is to provide rigorous and comprehensive information openly on rare diseases that all stakeholders can access. Orphanet nomenclature on rare diseases (ORPHAcode) ensures visibility of rare diseases in health and research information systems. As a consortium of 40 countries, within Europe and elsewhere, this unique initiative has a wide reach (see below).

In 2002 the United States Congress passed another law, 107/280, the Rare Disease Act (RDA), that also promises lasting effects - indeed influential beyond the immediate economic reach of the health regulatory systems and economies of the West. While the ODA accelerated therapeutic development by companies, it did not generate the necessary infrastructure within clinical practice and scientific research. This is needed outside industry to coordinate research and introduce policies that would support discovery population sciences and public health measures as well as education. The RDA led to Federal establishment of the Office of Rare Diseases to recommend a national research agenda, coordinate research that supports it - and provide educational activities for researchers. To foster collaboration and data-sharing between investigators and patient support groups, substantial funds were invested in the support, under the aegis of the National Institutes of Health, a national Rare Diseases Clinical Research Network. This comprises Rare Diseases Clinical Research Centers and a Data and Technology Coordinating Center - all designed to increase collaboration and data sharing between investigators and patient support groups. The overall aim is to improve the lives of those affected and ultimately prevent or eliminate these diseases. European Reference Networks also represent an impressive initiative by which to systematize clinical practice and improve access to expert opinion.

## Global perspective

Beyond what has been described, the pharmaceutical revolution has brought in train more nuanced and general benefits. The World Health Organization has adopted the concept though the International Classification of Disease (ICD). The latest version, endorsed by the World Health Assembly, came into effect on 1 January 2022. A further outcome is that the concept of rare disease has at least found acceptance across many countries of mixed wealth and stability and in politically divergent jurisdictions. Health statistics reported in the ICD-11 record health and health-related conditions; they ensure mutual compatibility of digital health data and comparability. In collaboration with Orphanet, WHO reviews the identity and coherence of about 5,500 rare diseases–and these activities are linked to the WHO Collaborative Global Network 4 Rare Diseases (WHO, 2022).

Increasing recognition of rare diseases worldwide and the potential consequences for expenditure on health care, disease management and diagnostic services has driven politicians to introduce new health policies. Many might suppose that this reflects the need to secure provision of expensive drugs or even highly expensive drugs. In general, however, the activity is independent of the orphan drug legislation and high-cost therapies. Adoption of specialized services does not necessarily raise demand for ultra-orphan treatments at exorbitant prices (>$100,000 annually).

With the encouragement of WHO, across the world, resource-poor, low- or middle-income countries have explored the frequency and burden of rare diseases. Widely dispersed regions with different jurisdictions and diverse health care system of provision have followed the sound principles of collecting information, securing knowledge and estimating the scale of the challenge. One should not forget that most rare diseases have a strong genetic cause and Mendelian conditions are highly overrepresented in this category. There are thus striking differences, almost unconscionable in their range, in the frequency of rare diseases between certain populations. There is a strong association with consanguinity related to cousin marriages as in parts of the Indian subcontinent, the Middle East, Arabian Peninsula, Eastern Turkey as well as North Africa (Matalonga et al., 2020). There are few glib or immediate solutions where social customs and cultural practices remain part of history and tradition but early clinical engagement with leading members of affected communities can be decisive. Naturally, given the resources required to deliver appropriate clinical and diagnostic services in widely differing jurisdictions, the pace of innovation will be heterogeneous and regionalized.

## Alignment and variation

While much has changed, there is a marked lack of consistency in methodology and non-uniform definitions of rare disease prevalence. In parts of Turkey, in Iran, Egypt and several other Middle-Eastern countries, including until recently Lebanon, the aspirations and reality reflect strong efforts to adapt services to meet the requirements for specialist centre provision. Populous nations such as India and China have undertaken in-depth reviews. For the Government of India, in 2017 the Ministry of Health and Family Welfare formulated a national policy for treatment of rare diseases. Implementation faced challenges based on marked interregional differences between the States of this enormous country and as explained: ‘lack of clarity on how much Government could support in terms of tertiary care’. Given the scale of the problem, the massive size of the country and the extremes of rich and poor without an effective public system for health, very few, except those with private fortunes, obtain adequate healthcare.

In Russia (Volgina and Sokolov) and China (Liu et al.), service provision is accommodated in specialised service centres and systematic referral practices have been introduced (as reflected in several high-quality contributions to this volume). It is notable however, that globally there remains a striking lack of cohesion in perceptions about rare diseases, their frequency and, once identified, the burden that they represent. WHO defines a rare disease as a disorder with a population incidence in the range of 0.65–1%. In China, a rare disease is one with a prevalence less than 1/500,000 of the population or, in the newborn, with a frequency of less than 1/10,000. It is not difficult to imagine that even at these defining limits, millions of patients with rare diseases will live in China and the consequences for the provision of specialised medical care would present a massive challenge for public health services.

### Protecting exclusivity

The issue of previously approved high-cost therapies for rare and ultra-rare diseases late in the aftermath of the Orphan Drug legislation introduces perhaps the most desirable outcome of the marketing exclusivity and ultra-high costs: the elapse of time. The period of exclusivity passes–after 7 or 10 (occasionally extended to 12) years. For exceptionally expensive orphan therapies (often molecular therapies such as recombinant proteins and monoclonal antibodies), the stimulus to develop competitors is strong where there is transformational efficacy. There are now three approved macrophage-targeted enzyme therapies for Gaucher disease. The pricing is high, very high but compared with costs of these therapies when first introduced 30 years ago, the annual cost is declining as, within limits, competition bites. However, generic recombinant proteins that are rigorously validated and safely prepared with reliable manufacturing and supply chains, are not easy to guarantee at sustainable competitive prices (Drelichman et al., 2020). Such practicalities do not always deter state-funded initiatives or even governments from breaching patents. Enzyme therapies for Gaucher disease developed in South Korea and Russia are, or have been used also in a few other countries including Iran and Mexico. Gaucher disease appears to be a special competitive case because the patent protection for the first-line oral substrate synthesis inhibitor drug approved in 2014–5 for Gaucher disease is subject to challenge in non-Western economies. Compared with small molecules that are readily synthesised and pirated, international Biotech companies generally prefer biologics since their global protection of approved Advanced Medicinal Therapies can be more readily validated - thus securing long-term marketing exclusivity.

## Regional matters

The needs of patients remain at the heart of the powerful initiatives that brought about the rare disease legislative frameworks but as reflected in several articles in this volume, many countries have yet to adopt the initiative so that provision of healthcare does not follow the US or European models. Patients in many places cannot obtain support for effective orphan drugs approved only and available only elsewhere. Lack of conformity for the development and marketing of lucrative orphan drugs (typified by the “Western way of thinking”) includes: 1) variable definitions of rare diseases by frequency across countries; 2) methods for estimating frequency or prevalence differ widely in accuracy and records may be non-existent in some jurisdictions; 3) the burden of care related to any particular disease differs markedly between regions. Sickle cell anaemia is endemic in large regions of Africa, parts of India and the Middle-East and affects an estimated 5 million persons. However, the same disease meets the definition of rare, even ultra-rare, in much of Europe and the Americas (excluding the Caribbean). Simply considering this one disease, the healthcare needs of patients with sickle-cell disease vary from very small to a major burden on the national economy. Quite apart from the extreme divergence across populations, the actions and legal policies adopted for managing rare diseases also differ radically. As Luzzatto and Makani (Luzzatto and Makani) point out, a relatively cheap drug, hydroxyurea (hydroxycarbamide), will offer relief for most of those that receive it but it is under-used in Africa. Unlike molecular therapies and the development of corrective gene transfer procedures in late clinical development for major Western hospitals, what is needed in Africa is support for centres and education of healthcare personnel to be able to estimate demand and organize services for delivery and monitoring–for example hydroxyurea therapy.

## Does venture capitalism belong in the field of rare diseases?

Quick profits, hard stopping points and selling on, do not seem to be a responsible way to manage a promising therapeutic programme in the face of the disadvantaged potential trial population suffering from a rare disease and with grief not far from the human surface. Politics is the art of the soluble and it is clear that for much of the world, access to highly specialised therapies through the agency of the Orphan Drug Act and follow-on legislation is inequitable. Patients denied access on financial grounds often would have been seen as placing unsustainable charges on national budgets that few systems can meet. African populations, who have endured the ravages of colonialism and European economic plunder over centuries, represent a stringent moral testing-ground for our sense of fairness (Luzzatto and Makani). The continental landmass of Africa, from the time of the slave trade, has hardly benefitted from the riches and benefits of industrialization, even though it has been plundered for raw materials and slave labour extracted from millions of its transported inhabitants. By nearly every measure, most modern African countries are grossly under-resourced for healthcare: according to international Organisation for Economic Co-operation and Development and World Bank (2021), 44 of all 54 independent African states remain in the lower middle-income category ($1,036 and $4,045 gross national income per capita); 7 are upper middle-income economies (GNI per capita between $4,046 and $12,535). Equity of access to treatment and financial restitution for those in need would help to solve the Marxian dilemma of what to do in health when capitalism fails.

At such times, the need to regulate gaming by companies and pricing beyond reasonableness within the capitalist system urgently mandates redress and action. This is now happening: venture capitalists are now more interested in licensing new technologies than for example ‘me too’ gene transfer with current vector systems. Alternative funding mechanisms for drug development and reimbursement should be agreed in advance for orphan agents - rather than allow manufacturers themselves to determine their charges. Agreed research consortia, as with the Cystic Fibrosis Foundation, that led to the identification and trial of exceptional, small molecules with excellent tolerability and striking efficacy for this scientifically challenging disease (Abdallah et al.) are the invention of resourceful charities. Even in rich countries, financial markets are now showing their displeasure at what might be seen as exploitative self-interest for high-charge therapies in financially privileged environments. Even if near-cure appears to be in reach, exorbitant costs of some molecular therapies such as the one-off and much-feted gene therapy, Zolgensma™ (onasemnogene abeparvovec-xioi) used for young children with spinal muscular atrophy, are questioned. If more patients are to benefit from the 40 years of activity since the Orphan Drug Act and 20–25 years since the Orphanet initiatives alongside the Rare Disease Act, then wider societal thinking and informed public debate is required. We need also to take stock of all drugs for a rare disease to ensure that the effectiveness of any high-cost therapy is thoroughly understood, explored - and scored according to whether it achieves clinically articulate outcomes. ‘Real-world’ evaluations that include verification of tolerability and effectiveness by the patients who receive a given agent are gaining credence. Concepts of ‘conditional regulatory approval’ and ‘payment by results’ for marketing and reimbursement are also taking hold.

We should recall that marketing exclusivity is granted not necessarily for the best drug nor necessarily what would meet the criteria for a good drug: the principal criterion is that the approval is given for the first safe drug to have *any* efficacy. The authors are well aware of some exorbitantly costly enzyme preparations for lysosomal diseases that are the only approved and attempted fix, but are neither life-saving nor more than minimally effective. It is as if there is a two-tier system: 1) care that constantly affects daily lives but involves stratagems which are biochemically and nutritionally straightforward and usually cheap; 2) highly specialised molecular agents, cell and gene therapies imbued with high expectation and exorbitant costs with complex delivery under specialist management. When considered against the need across populations, few drugs have yet proven to be transformational: most used for rare diseases are special diets (eg. low-protein, fructose-free, galactose-free) or require supplemental factors (eg. vitamins such as vitamin B1, biotin, pyridoxine/pyridoxal or the vitamins B12 and folic acid) and supportive care that is relatively inexpensive and affordable (Hendrickx and Dooms). A final point is that medical care for rare diseases constitutes far more than the ‘magic’ of the specific high-cost drugs: time-honored principles of clinical practice are paramount. By the same token, disease management is not the pedestrian application of “efficiency gains”. Rather it involves direct interactions with the patient and relief of symptoms specific to them and their disease. Combined with the primacy of serving as the patient’s advocate and attending to education – in part through genetic counseling – simple actions often have prodigious effects on life quality.

## Prospective

A prescription for the field itself, in many ways stimulated by the authors in this volume, is strategic discussion and dialogue with stakeholders worldwide. Rare diseases, like infectious disorders, are a collectively massive and comparable human burden. We can thus go further, perhaps best exemplified by the global action to combat HIV/AIDS. In the African region alone, this has had an immense impact and 5 years ago, patients in the Africa were able to gain access to life-saving treatments that represented more than two-thirds of the global HIV drug market. While emerging drug resistance has hampered achievement of the goal of widespread viral suppression, an internationally reinforced multifactorial approach is in the ascendant against this infection. To this, with the engagement of the Bill and Melissa Gates Foundation among other international charities, the conquest of tuberculosis has latterly been included (Bill & Melinda Gates foundation, 2022) alongside the leading WHO Global Tuberculosis Programme and End TB strategy (TDR, 2022).

Rare diseases harbor a distinct set of complexities but the crushing injustices reflected in the unequal provision of health care to treat them represent a unique challenge to the global political will. No one affected by the pressing needs of patients across all communities can afford to ignore the enormity of such disparities at a time of great social movement and revolution in the 21st century CE. It would, after all, defy the principle of ‘enlightened self-interest’ to do so.
